# p73, like its p53 homolog, shows preference for inverted repeats forming cruciforms

**DOI:** 10.1371/journal.pone.0195835

**Published:** 2018-04-18

**Authors:** Jana Čechová, Jan Coufal, Eva B. Jagelská, Miroslav Fojta, Václav Brázda

**Affiliations:** 1 The Czech Academy of Sciences, Institute of Biophysics, Královopolská, Brno, Czech Republic; 2 Department of Biochemistry, Faculty of Science, Masaryk University, Kotlarska, Brno, Czech Republic; Virginia Commonwealth University, UNITED STATES

## Abstract

p73 is a member of the p53 protein family and has essential functions in several signaling pathways involved in development, differentiation, DNA damage responses and cancer. As a transcription factor, p73 achieves these functions by binding to consensus DNA sequences and p73 shares at least partial target DNA binding sequence specificity with p53. Transcriptional activation by p73 has been demonstrated for more than fifty p53 targets in yeast and/or human cancer cell lines. It has also been shown previously that p53 binding to DNA is strongly dependent on DNA topology and the presence of inverted repeats that can form DNA cruciforms, but whether p73 transcriptional activity has similar dependence has not been investigated. Therefore, we evaluated p73 binding to a set of p53-response elements with identical theoretical binding affinity in their linear state, but different probabilities to form extra helical structures. We show by a yeast-based assay that transactivation *in vivo* correlated more with the relative propensity of a response element to form cruciforms than to its expected *in vitro* DNA binding affinity. Structural features of p73 target sites are therefore likely to be an important determinant of its transactivation function.

## Introduction

p73 is a member of the p53 protein family and is involved in processes including cell cycle regulation and apoptosis [1,2]. Due to sequence homology with the human tumor suppressor p53, p73 has been suggested to function in tumor suppression [3]. However, cancer development is rarely associated with p73 mutations, with possible exceptions being loss in a subset of T-cell lymphomas and neuroblastoma [4,5], and no genetic disorder has been linked to p73, in direct contrast to p53 [6]. It has been demonstrated that p73 plays important roles in cellular differentiation [7] and many human tumors including breast and ovarian cancer show an increased expression of p73 [8–10].

p73 exhibits 63% amino acid sequence identity with p53 in the DNA-binding domain [11]. Therefore, it is not surprising that p73 can recognize the same response element (RE) as p53 and activates an analogous set of downstream genes. Similarly to p53, p73 binds to DNA cooperatively as a tetramer and despite structural differences in the oligomerization domain, the dissociation constants of tetramers are in the low nanomolar range indicating that the strength of tetramerization was evolutionarily conserved [12]. Prediction of p53/p73 binding sites in the genome showed almost complete overlap [7], but there are also several examples of genes exclusively targeted by p53, p63 or p73 [13,14]. Similar to p53 and p63, p73 has several isoforms. For example, DNA-binding activity was increased by deletion of the COOH-terminal region of p73α [15]. Moreover, ΔTA-p73 isoforms act as dominant-negative inhibitors of p53 by competing for sequence specific DNA binding at p53/p73 REs [16]. Biochemical analyses show analogous DNA binding specificities for p53 and p73, even though quantitative differences for certain DNA sequences have also been reported [12,17]. These differences could be caused by several factors including various protein-protein interactions for the less conserved N- and C- terminal domains, as well as variations of chromatin structure [18].

The p53-family target site has a consensus RE consisting of two decameric half-sites that may be separated by a short spacer (n): RRRCWWGYYY(n)RRRCWWGYYY [19,20]. Complex *in vitro* analysis of all possible REs allows calculation of the theoretical binding affinity of any DNA sequence [21]. It was also shown that p53 can bind efficiently to superhelical DNA [22–24] and to different local DNA structures [25]. Moreover, cruciform structures within p53 target sites facilitate p53 binding to DNA [26–28]. Inverted repeats able to form cruciform structures are overrepresented in promotor and regulatory regions and they are also often targets for protein binding [29,30]. The crystal structures of all p53 family members show conserved DNA recognition residues [31–33] and there is a high degree of overlap in transactivation potential and specificity between full-length p53, p63 and p73 [34]. Therefore, we used a yeast isogenic system as a sensitive assay that identifies subtle changes in transactivation potential [19,35,36] to validate p73 binding properties to DNA with defined inverted repeats. Our results show that, similar to p53, p73 is more active on sites that are able to form cruciform structures within DNA target sequences and that p73-dependent transactivation is stimulated by cruciform structures with longer loops in the center of p73 target sites. Not only DNA sequence, but also its structure in chromatin therefore plays a significant role in p73 transactivation.

## Materials and methods

### Construction of cruciform structure models in p53 target sequences

We used mfold software to determine structure and free energy (dG) of local DNA structures formed in p53 target sites [37].

### Analysis of inverted repeats in p53-target sites

We used DNA analyser software [38]. The parameters of analyses were set from 7 to 10 bp, spacer size was set from 0 to 10 bp and maximally one mismatch was allowed. Analysis produced a separate list of inverted repeats found in each p53-target sequence–we provide information about CF (cruciform) rank in format: **L**ength/**S**pacer/**M**ismatch in Table 1.

**Table 1:** In silico analyses of p53-REs ranked by p73 transcription activation (TA ratio) compared to empty vector. Bases which form an inverted repeat are in bold. The presence of inverted repeats was analyzed by Palindrome finder [38] with parameters 7-10/0-10/0-1 (**L**ength/**S**pacer/**M**ismatch). CF rank in the same format is shown in the last column. TA ratios were derived from [32].

	p53 target	P53 target sequence	TA ratio	Inverted repeat	CF rank
	*Canonical site*	*RRRCWWGYYY-RRRCWWGYYY*	*p73*	*7bp and longer*	*7-10/X/0-1*
1	CON S	**GAACATGTTC-GAACATGTTC**	85.3	yes	10/0/0
2	CON C	GGGCAAGTCT-GGGCAAGTCT	65.3		-
3	mFAS	G**G**G**CATGT**AC-AA**ACATG**T**C**A	44.4	yes—with mismatch	7/4/1
4	MMP2	**AGACAAG**CCT-GAA**CTTGTCT**	38.3	yes	7/6/0
5	P21-5'	C**AACATGT**TG-GG**ACATGTT**C	36.9	yes	7/4/0
6	R2	T**GACATGCC**C-A**GGCATGTC**T	36.1	yes	8/2/0
7	PA26	GGACAAGTCT-CAACAAGTTC	32.4		-
8	CON A	**GG**G**CATG**TCC-GGG**CATG**T**CC**	26.6	yes—with mismatch	7/0/1
9	PUMA	CTGCAAGTCC-TGACTTGTCC	24.3		-
10	MDM2-P2C	GGTCAAGTTG-GGACACGTCC	16.8		-
11		GAGCTAAGTCcTGACATGTCT			-
12	miR-34a-RE1	GGGCTTGCCT-GGGCTTGTTC	14.8		-
13	CON E	GAGCATGTCC-GAGCATGTCC	13.3		-
14	CON L	**G**G**GCATGC**TC-GG**GCATGC**T**C**	12.9	yes—with mismatch	8/0/1
15	BAX A+B	TCACAAGTTAgAGACAAGCCT	11.5		-
16		AGACAAGCCT-GGGCGTGGGC			-
17	miR-202	GGGCATGTCC-TGGCAAGCCT	8.7		-
18	hFAS	TGGCTTGTCA-GGGCTTGTCC	7.6		-
19	P21-3'	GAAGAAGACT-GGGCATGTCT	7.0		-
20	GADD45	GAACATGTCT-AAGCATGCTG	7.0		-
21	KILLER	GGGCATGTCC-GGGCAAGACG	6.4		-
22	p21 S2	GAACAGGTCC-CAACAGGTTG	5.7		-
23	RGC	GGACTTGCCT-GGCCTTGCCT	3.6		-
24	CYCLIN G	AGG**C**T**TGCCC**-**GGGCA**G**G**TCT	3.6	yes—with mismatch	7-0-1
25	AIP1	TCTCTTGCCC-GGGCTTGTCG	2.7		-
26	NOXA	AGGCTTGCCC-CGGCAAGTTG	2.2		-
27	miR-221	GAACATGCAT-GCACATGTTT	1.7		-
28	miR-198	AGGCAAGCTT-CAACAAGCCG	1.6		-
29	PAI	ACACATGCCT-CAGCAAGTCC	1.6		-
30	XPC	GGGCATGGTG-GCACATGCCT	1.6		-

### Theoretical p53 binding affinities

Theoretical p53 binding affinities were calculated by “p53 binding predictor“, an on-line tool use algorithm developed by Veprintsev and Fersht [21] which is freely available (http://bioinformatics.ibp.cz/#/en/p53-predictor).

### DNA

Supercoiled plasmid DNAs of pBluescriptIISK(-) and derived plasmids pCFNO [24], pB-XA, pB-TT, pB-XG, pB-GCG, pB-XT and pB-WC were prepared by cloning the oligonucleotides (XA, TT, XG, GCG, XT) with *Hind*III adapters into the *Hind*III site of pBluescript, Plasmids were purified from *E*. *coli STBL4* strain using ZymoPURE Midi Prep Kit and verified by Sanger sequencing. All plasmid sequences are provided in the multiple FASTA format (S1 file).

### Detection of non-B DNA structures in plasmids by S1 nuclease cleavage

2 μg of plasmid DNA was digested with S1 nuclease for 2 hours at 37°C in S1 nuclease buffer, precipitated in ethanol, dissolved in water and digested with *Sca*I for 1 hour at 37°C before separation by electrophoresis on 1% agarose gels.

### Yeast strains

We used a panel of *S*. *cerevisiae* haploid reporter strains (yLFM-REs); all strains are isogenic except for the different p53 REs located upstream of the luciferase reporter gene. The targeting of p53 target sequence of interest by the replacement of the ICORE cassette, using transfected single strand oligonucleotides, was performed following the *Delitto Perfetto* technique [24]. Correct targeting events were isolated exploiting the counter-selectable and the reporter selection markers of the ICORE cassette and confirmed by colony PCR across the modified locus and Sanger DNA sequencing.

### Yeast based luciferase assay

Yeast cells were grown in 1% yeast extract, 2% peptone, 2% dextrose with the addition of 200 mg/L adenine (YPDA medium). yLFM isogenic derivative yeast strains constructed for this study were transformed with three different plasmids: pTSG-empty (control vector), pTSG-hp53, or pTSG-hp73 (for the expression of wild-type human p53 or p73 under the inducible *GAL1* promoter). These plasmids are based on the centromeric vector pRS314 and contain the *TRP1* selection marker [23]. Luciferase was measured using Bright-Glo^TM^ (Promega), as previously described [25].

### Statistical analysis

Transactivation data are plotted as fold induction of luciferase activity relative to the reporter activity measured with cells that contain pTSG-empty plasmid cultured under the same conditions. Mean and standard deviation of at least three biological replicates are presented. Statistical significance was evaluated using Student’s t-test. Statistical evaluation of p73 transactivation ratio in the sequences without and with inverted repeats were performed by Wilcoxon rank test with continuity correction.

## Results and discussion

### Correlation of p73 affinity and in silico analyses of p53-REs for the potential to form cruciform structures

**Fig 1:**
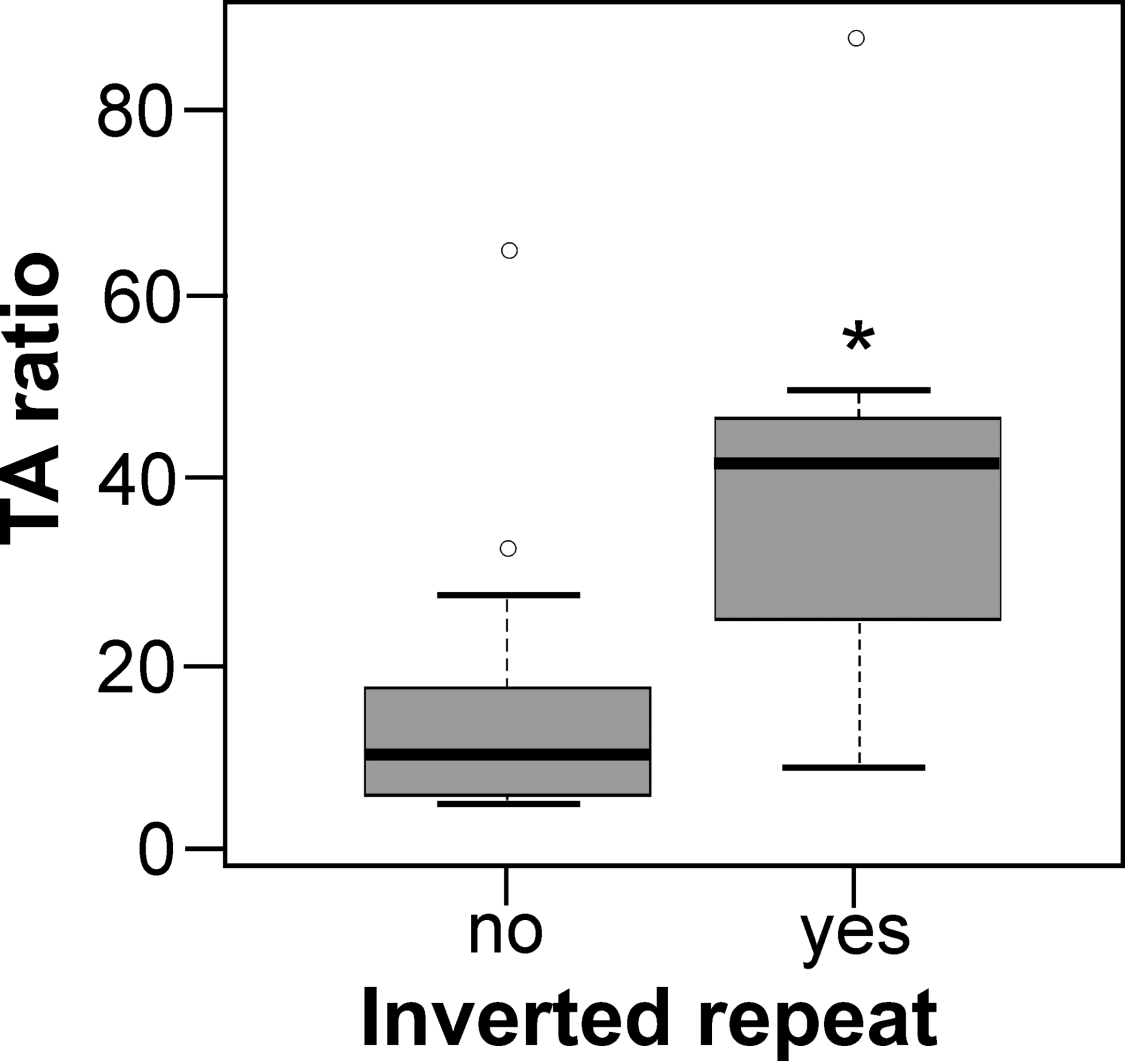
Comparison of the p73 transactivation ratio in sequences without (left) and with (right) inverted repeats–a box plot (data from Table 1). Comparison by Wilcoxon rank test with continuity correction show significantly higher values for the selection with the inverted repeats at target sites (p<0.01), marked with an asterisk.

A recent paper “Transactivation specificity is conserved among p53 family proteins and depends on a response element sequence code” [34] showed that p53, p63 and p73 share similar binding affinity and overlapping transactivation profiles for a considerable number of DNA targets in yeast and human cell lines. *In silico* analysis of the presence of inverted repeats in the sequences used in this study shows that most sequences with high transactivation activity correspond to those with possible cruciform structure formation (Table 1). Comparison of the p73 transactivation ratio in sequences without and with inverted repeats by Wilcoxon rank test with continuity correction show significantly higher values for the selection with the inverted repeats (Fig 1).

**Fig 2:**
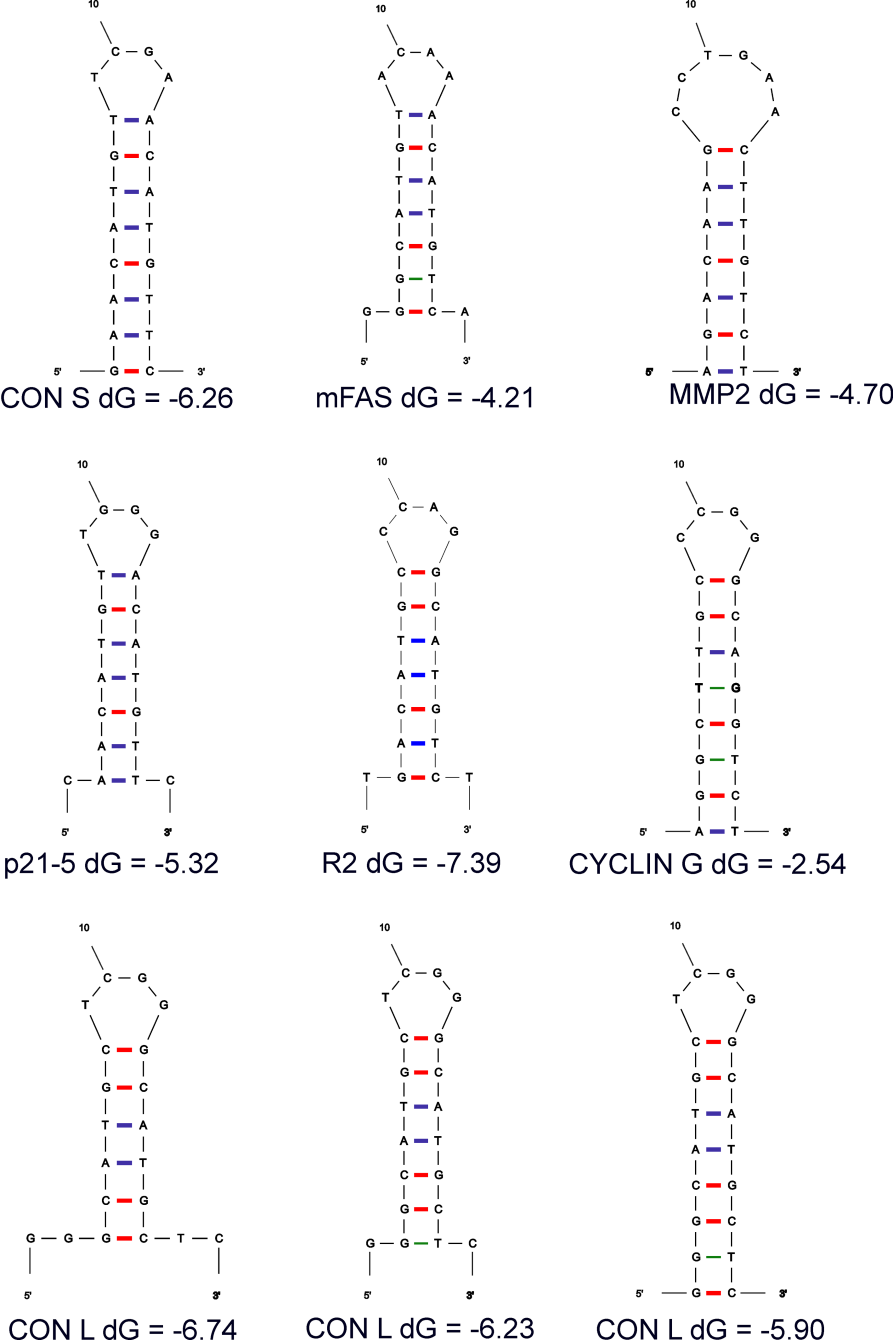
Models of cruciform structure formation in p53 target sequences. Using mfold software we analyzed the structure and dG of the indicated p53 target sequences with potential to form cruciform structure (see Table 1). GC bonds are shown in red, AT bonds in blue and mismatched GT bonds in green.

We used mfold software to predict the potential structure of p53 target sites in these sequences (Fig 2). The mfold software identified the most favorable structures and the dG energy of these structures [37]. All p53 target sequences that are efficiently bound by p73 have an inverted repeat located on the edge of the target sequence and most bases in the cruciform are ideal Watson-Crick pairs (G-C, shown in Fig 2 by red, or A-T, shown by blue). Only the Cyclin G target sequence with relatively low transactivation potential compared to other DNA targets, has two mismatches in the stem part of the cruciform (pairs C-T, shown in Fig 2 by green). These mismatches are the main reason for higher dG of this sequence, which makes the formation of the cruciform structure in this sequence less probable and less stable compared to the other target sequences with high p73 transcription activation. Interestingly, mfold suggested formation of three different loops in the CON L p53 target sequence (Fig 2, third row). However, all these structures had a lower theoretical dG than the Cyclin G sequence suggesting higher probability of formation and stability of cruciform structure.

### Comparison of p53 and p73 transactivation in yeast cells

We have previously shown that transactivation *in vivo* correlated more with relative propensity of the DNA target to form cruciforms than to its predicted *in vitro* DNA binding affinity for p53 and that structural features of p53-REs could therefore be an important determinant of transactivation by p53 [28]. To analyze p73 transcription activity on p53 target sequences, we used isogenic yeast that differ only in the p53 target sequence to compare p53 and p73-dependent transactivation from a luciferase reporter gene placed in a specific chromatin context. The p53 target sequences were cloned upstream of the luciferase gene at the *ADE2* locus and we analyzed transactivation induced by p73 protein controlled by the *GAL1* promoter. We used three yeast isogenic constructs–empty without p53 target site, and two constructs XA and XG with p53 target sites. These constructs are based on the common CATG sequence in the center of the REs, seen in many natural p53 binding sites (mFAS, p21, Gadd45 and others; see Table 1) and were designed to represent an idealized testing system for modification of individual flanking nucleotides without the interference of alternative sequences in endogenous p53 REs.

**Fig 3:**
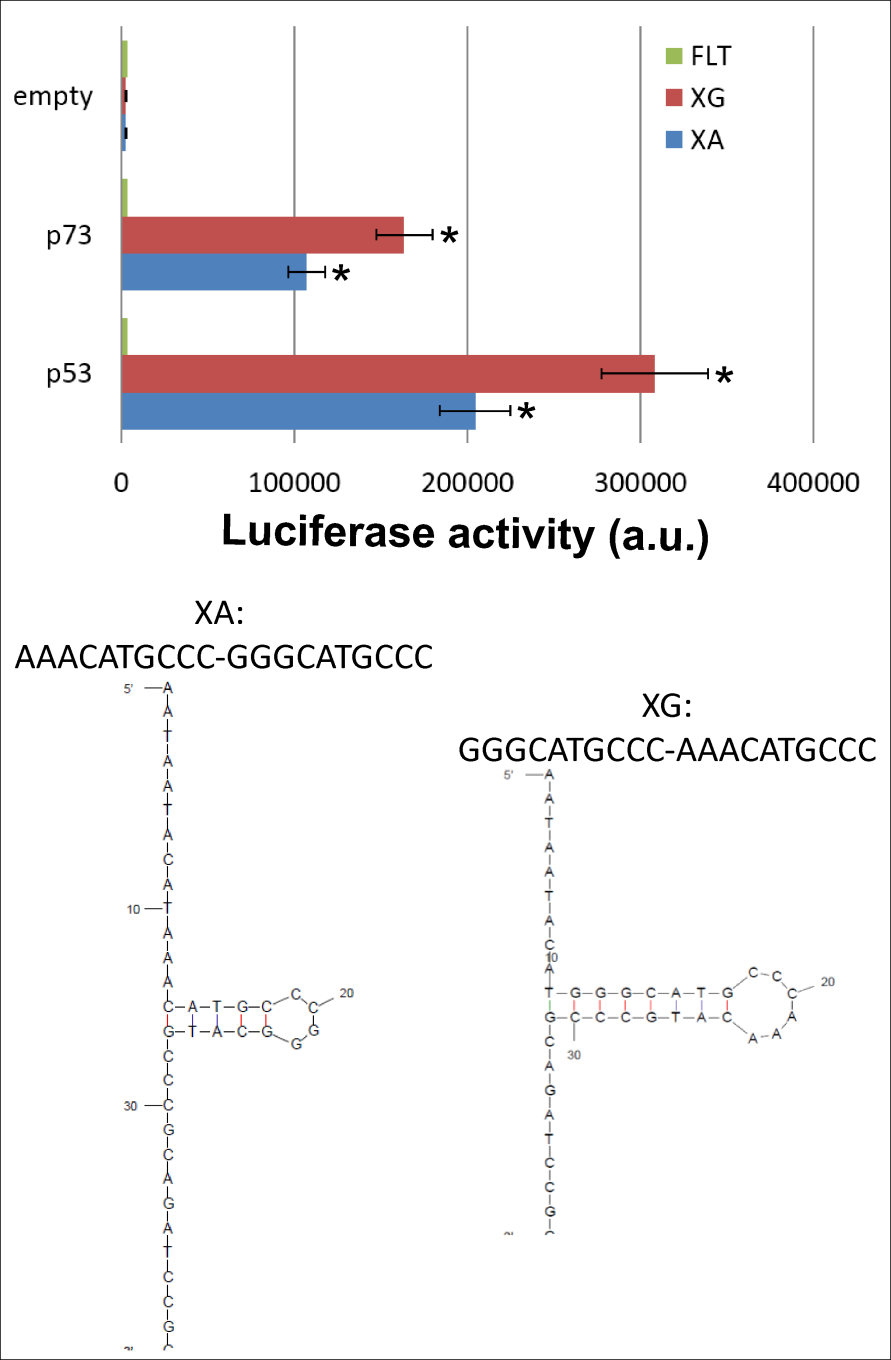
Comparison of p53 and p73 transactivation in yeast. Three isogenic yeast strains were used, two with p53 targets sites (XA and XG) and one without a p53 target site (FLT) upstream of the luciferase gene. Cells containing pTSG-p53 (p53, left), pTSG-p73 (p73, middle) or pTSG with no insert (empty, right) were treated with galactose to induce p53 or p73 from the *GAL1* promoter. The histogram plots average luminescence and standard deviations of three biological replicates. Asterisks indicate a significant induction of p53 or p73-dependent transactivation (p<0.05). The sequences of the XA and XG constructs are shown in their potential cruciform structures.

The experimental constructs differ in the location of their A and G tracks in the flanking sequences. While XA has an A-track located at the edge of the p53 target sequence and a G-track in the middle of the target sequence, XG has opposite locations of these tracks. Both sequences are p53 targets, but form different structures according to mfold; formation of a small structure with 4 bases in the stem of the cruciform is predicted in XA, while XG could form a longer and more stable structure with 7 base pairs in the stem of the cruciform (Fig 3). We compared transcription activation by p53 and p73 *in vivo*. Results show very low transactivation using the construct without a p53 target site (Fig 3, empty), with high signals for both p53 and p73 in yeast containing p53 target sequences. p73 transactivates both p53 targets efficiently, although transactivation is significantly higher in cells expressing p53 than p73. For both proteins we observed significantly higher transcription activation for XG compared to XA target sequences. These results suggested that p73 binds to p53 target sequences in an analogous manner to p53.

### p73 transactivation differs in yeast cells not only according to theoretical binding affinity, but also according to predicted structure in response elements

**Fig 4:**
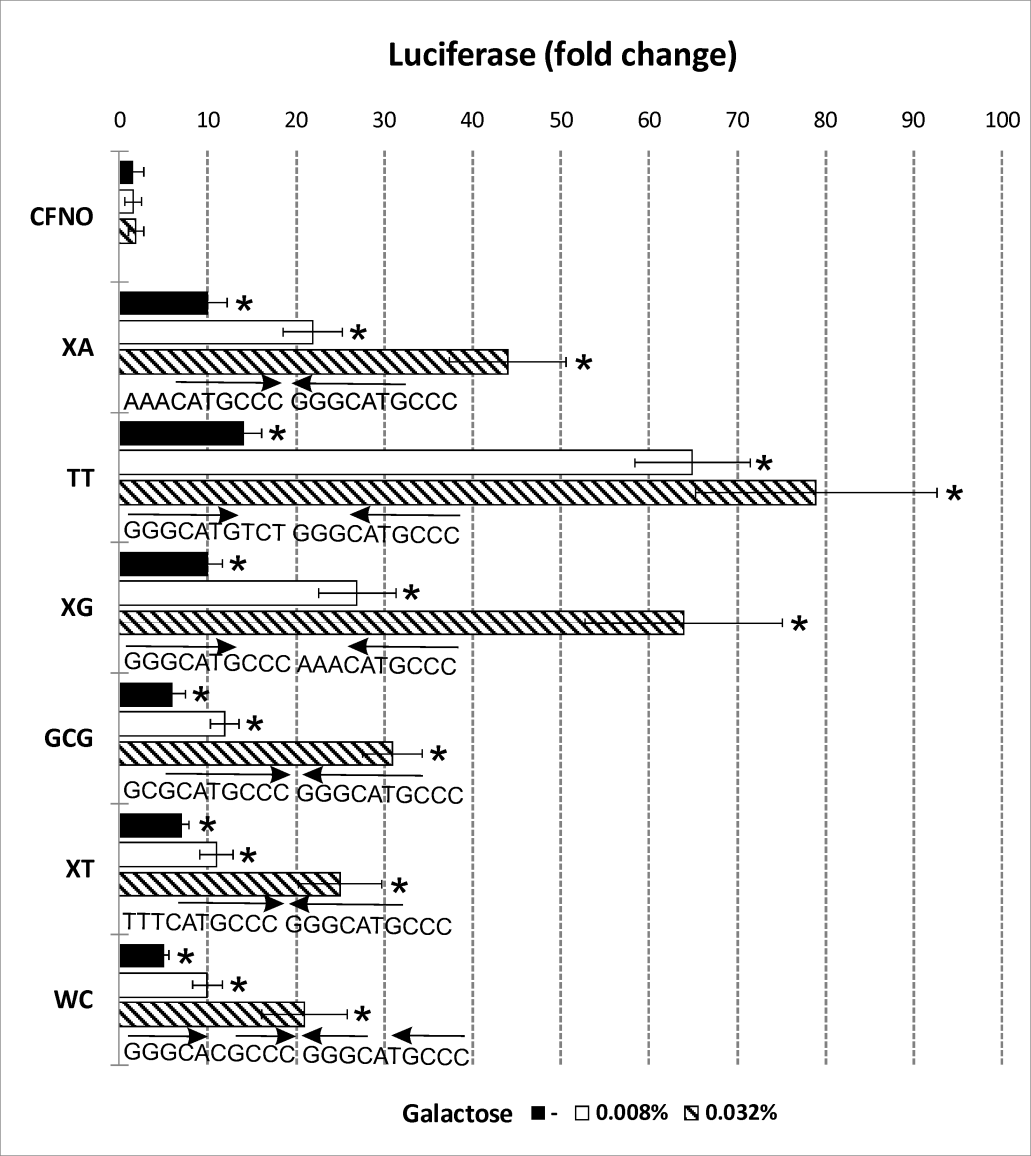
p73-dependent transactivation potential in yeast. p73 protein was expressed from pTSG-p73 under an inducible *GAL1* promoter. The indicated reporter yeast strains were also transformed with an empty pTSG vector and reporter activity was normalized to cell numbers and plotted as fold induction over empty vector. Average luminescence and standard deviations of three biological replicates are shown. For each strain, luciferase activity was measured at 6 hours of culture in media in the absence of galactose (black) or after induction of p73 with two different concentrations of galactose to induce different levels of p73 (0.008% galactose, white; 0.032% galactose, streaked). Asterisks indicate significant induction of p73-dependent transactivation at each galactose level (p<0.05). RE sequences are shown below each set of conditions.

To analyze p73 transcriptional activation in more detail, we used a set of 6 yeast isogenic strains with p53 target sequences that differ in their theoretical p53-DNA binding affinities [21] and have different propensities to form cruciform structures. As a control, we used a construct with an ideal inverted repeat but without a p53 target sequence (CFNO). Each pair of the set (XA and TT– ΔlogKd 0.08, XG and GCG– ΔlogKd 0.18, XT and WC– ΔlogKd 0.32) has identical theoretical p53 binding affinity, but differ in the location and quality of the inverted repeat. Therefore, if formation of a cruciform does not influence p73 transactivation, transactivation will be similar for pairs with identical theoretical binding affinity. We assessed the formation of cruciform structures of all tested sequences cloned in plasmid DNA by S1 nuclease cleavage. pB-TT and pB-XG plasmids showed preferential cruciform formation in the p53 target sequence at native superhelix density. As expected, we did not observe p73 induced transactivation for the CFNO construct that forms a cruciform but lacks a p53 target site (Fig 4, first bars) in yeast based luciferase assay. Transformation of the isogenic yeast strains by pTSG-hp73 and induction of p73 protein with galactose led to a significant increase of transcriptional activation for all constructs with p53 target sites. In general, the level of activation corresponded to theoretical p53 binding affinities. When we averaged the transcription activity for both p53 targets with the same theoretical DNA binding affinity, the best p73-induced transactivation was for XA and TT constructs, followed by XG and GCG and the lowest activation was observed for XT-WC constructs. However, the transcription affinities differ significantly within the first two pairs. Moreover, the transcription activation of the XG construct with lower theoretical DNA binding affinity is significantly higher than the XA construct that has higher theoretical DNA binding affinity. Interestingly, we also observed significant differences within groups with the same theoretical DNA binding affinities. The best transactivation occurred with TT, which has identical theoretical DNA binding affinity as XA, but the XA construct has the inverted repeat located in the middle whereas the TT construct has the inverted repeat located at the edge of the sequence. This feature leads to better cruciform propensity and different location in the structure. Similarly, the XG sequence, with lower theoretical p53-DNA affinity compared to XA and TT constructs, has an identical inverted repeat as TT and has significantly higher transcriptional activation compared to its paired GCG construct. The differences in the level of transcription for XT and WC constructs were not as great as for the first two pairs. This suggests that that combining an inverted repeat in the middle (XT) with disruption of the inverted repeat (WC) leads to less probability of cruciform formation and lower transcriptional activity of p73 protein compared to TT and XG targets.

## Discussion

p73 is considered as a pharmaceutical target for cancer therapy due to its upregulation in several malignancies [8–10,39]. The sequence similarity of the DNA binding domain in p53 family proteins compared to the diversity of other regions of p53, p63 and p73 suggest that the evolutionary conserved shared regions are important parts for DNA binding in all family members [40]. For example, the cysteine residues in the core domain of p53 family proteins are conserved and their oxidation abolishes sequence-specific binding [41]. Similarly, heavy metals have equivalent effects on the conformation of p53 and p73 and on the binding of their core domains to DNA [42]. Thus, the regions involved in the direct binding to DNA are probably identical in the three family members, whilst the diverse C-terminal domains of these proteins modulate DNA binding and transcription activities [43]. Due to amino acid sequence identity reaching 63% in the DNA-binding domain, it was proposed that p53, p63 and p73 should have redundant functions in the regulation of gene expression [11]. On the other hand, TA isoforms of p63 differ in their transcriptional activities toward genes regulated by p53, and while TAp63gamma is the most active form, DeltaNp63 isoforms are transcriptionally inactive and inhibit TA isoforms [44].

The influence of systematic variations in the target sequence on the binding affinity of p73 has been reported. The largest determinant of DNA binding was the cytosine in the fourth position of each quarter-site, followed by the nucleotide in the fifth position, and last, the first three positions show a slight regulatory preference for purines [45]. Those results showed that some nucleotide positions in the response element are more important than others in determining the binding of the transcription factor [45]. The findings are in agreement with our results, where the CATG sequence in the middle of the response elements is crucial for effective p73 binding to DNA. Moreover, this sequence of response elements could be part of the inverted repeat which can even enhance the effectivity of the p73 transactivation as a result of its binding to DNA. It has been demonstrated that mutant p53 blocks DNA binding and transactivation by p73 [46]. Combinations of the different regulatory pathways could therefore be important for distinctive regulation in particular cancers. Inverted repeats and SNPs leading to improvement or abolishment of cruciform propensity in regulatory elements could be therefore an important factor with potential therapeutic and pharmacological utilization. The presentation of the target site in a cruciform structure could lead to a more effective and/or more stable protein-DNA complex, leading to increases in both protein and DNA structure stability. It has been shown that some p53 mutants can bind DNA and adopt a wild-type conformation in vitro but are transcriptionally inactive in vivo [47]. Our results show not only formation of cruciform structure in plasmid DNA and p73 binding in vitro, but also that p73 is capable of transcriptional activation in these sequences in chromosomal DNA in vivo.

In addition to several factors influencing protein-DNA binding and the precise DNA sequence of the target, local DNA structures play important roles in basic cellular processes [48,49], including influencing sequence-specific p53 binding and transcriptional activation. It was shown that not only cruciform structures, but also the general opportunity to form non-B structures in p53 responsive sequences improved its binding [24,28]. Moreover, p53 is only one of many proteins to show preferential binding to cruciform structures [29,50]. The correlation between p73 protein transactivation activities with the presence of inverted repeats in our analyses leads to the question: Is p73 another protein with preference to inverted repeats and cruciform structures? Our results show that the presence and location of the inverted repeat changes p73 transcription efficiency in isogenic yeast. Therefore, we can conclude that p73 binds to p53 target sequences not only according to sequence but also according to structural features, similar to p53 binding to DNA. Therefore, DNA sequence is not the only determining factor for p73 transactivation in a chromatin context. These notable features of p73 binding to structured DNA are likely to be an important aspect of the complexity of p73 regulated pathways.

## Supporting information

S1 File Sequences of plasmids used in the study in the multiple FASTA format. (FASTA)
